# Clear Cell Hidradenoma: A Review of Reported Cases

**DOI:** 10.7759/cureus.101508

**Published:** 2026-01-14

**Authors:** George Ladas, Christopher Stewart, Peter A Khoury, Adam Kramer, Charles Harper, Pear Sukarom, Niritta Patel, Patricia Palanca, Gursuraj Grewal, Suporn Sukpraprut-Braaten

**Affiliations:** 1 General Surgery, Kansas City University, Joplin, USA; 2 General Surgery, St. Mary's Medical Center, Blue Springs, USA; 3 General Surgery, University of California Los Angeles, Los Angeles, USA; 4 Graduate Medical Education, Kansas City University, Joplin, USA

**Keywords:** cancer, clear cell hidradenoma, dermatology, hidradenoma, interdisciplinary care

## Abstract

Clear cell hidradenoma (CCH) is an extremely rare benign adnexal skin tumor originating from eccrine sweat glands. The purpose of this systematic review is to analyze and demonstrate trends in the location, occurrence, and presentation of CCH as well as its ability to present in advanced disease states. This study identified 460 case reports published from 1979 through 2022 through PubMed searches. Preferred Reporting Items for Systematic Reviews and Meta-Analyses (PRISMA) guidelines were tightly adhered to. The search keywords were “acrospiroma” and “clear cell hidradenoma.” Investigators reviewed the articles and extracted the following information: demographics; medical history and medications; anatomic location and characteristic of the CCH; malignancy; and CCH progression timeline. Of the 460 cases reported, 228 reports with 241 cases of CCH were included in the final analysis. The average age is 54.7 (±21.1) years old, and 54.36% are females. Thirty-five CCH cases were reported to be malignant. The genitourinary system is the most common location for malignant CCH. No statistically significant difference in malignant transformation was observed between genders (p=0.24). Malignant CCH had a mean length 2 cm greater than nonmalignant CCH (p-value=0.023). Cases with reported malignant CCH had CCH for a mean of 2.75 years longer than nonmalignant CCH cases (p-value=0.048). CCH is a rare skin tumor commonly found in genitourinary, facial, and head areas. No significant gender difference was found when looking at malignant CCH. The length of the CCH and the time it grows could be indicators of malignant CCH. Careful histopathologic examination, biopsy, and surgical excision of the tumor are recommended, although there is a chance of recurrence.

## Introduction and background

Clear cell hidradenoma (CCH), also known as nodular hidradenoma or solid cystic hidradenoma, is a rare benign skin tumor originating from eccrine sweat glands that can be seen in the head, neck, and extremities [1]. It predominantly affects adults and has been reported more frequently in females [1,2]. CCH is characterized by reddish-purple areas and linear hairpin-like vessels on the surface of the tumor [1]. CCH is molecularly characterized by the t(11:19)(q21;p13) TORC1-MAML2 gene fusion, a rearrangement also identified in mucoepidermoid carcinoma and Warthin’s tumor, suggesting shared molecular pathways for tumorigenesis [3]. CCH may recur and present similarly to other clear cell tumors, leading to misdiagnosis [4]. This underlies the importance of careful histopathologic examination [1,5]. While Hernández-Pérez et al. (1985) reported a 10-year review of CCH cases in 1985, no comprehensive contemporary synthesis exists that integrates both malignant and nonmalignant cases across the modern literature; this systematic review aims to address that gap [6].

## Review

Materials and methods

Information Sources and Strategy

This systematic review was performed in accordance with the Preferred Reporting Items for Systematic Reviews and Meta-Analyses (PRISMA) guidelines. The literature search was conducted using PubMed due to its comprehensive indexing of biomedical case reports; however, additional databases, including Embase, Web of Science, and Scopus, were not searched. Following study selection, included cases were independently reviewed, and demographic and clinical variables were extracted, including age, sex, anatomic location, and histologic classification (benign versus malignant). Cases were grouped according to malignant status, and comparative statistical analyses were performed to evaluate differences between benign and malignant CCH. Data extraction and categorization were performed by the study authors, with discrepancies resolved by consensus.

Eligibility Criteria

Non-case report articles (including reviews, editorials, and commentaries) and articles not published in English were excluded. Included case reports were required to meet the Joanna Briggs Institute (JBI) critical appraisal criteria for case reports and to provide sufficient clinicopathologic information to confirm a diagnosis of CCH, including patient demographics, lesion location, and histopathologic findings [7].

Study Selection

**Figure 1:**
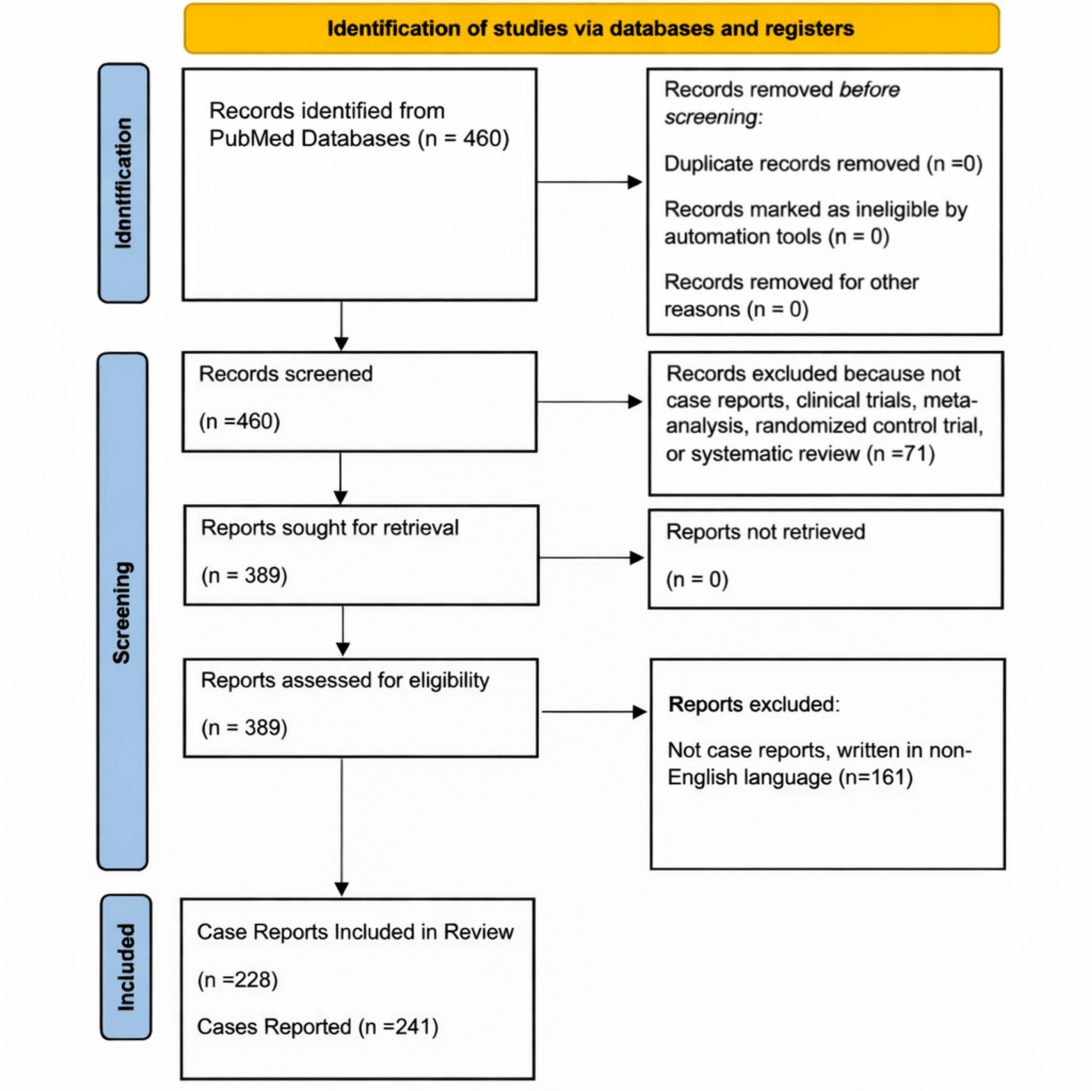
Preferred Reporting Items for Systematic Reviews and Meta-Analyses (PRISMA) flow diagram

Articles were obtained from the PubMed database. A systematic literature search was conducted on February 15, 2022, using the following Boolean query: (‘clear cell hidradenoma’ OR ‘acrospiroma’), applied to the title and abstract fields. All articles published between 1979 and 2022 were retrieved, yielding 460 records. Subsequent filtering was performed by removing non-case report articles. Further filtering excluded non-human studies and non-English publications. After screening and eligibility assessment, 228 case reports comprising 241 individual cases were included in the study. The discrepancy between the number of reports and cases reflects that a small number of publications described more than one case, whereas the majority reported single cases. Each reported case was extracted and analyzed individually. Given the descriptive nature of the analyses, potential clustering of cases within individual reports or institutions was not adjusted for and is acknowledged as a limitation. The PRISMA guidelines were strictly followed, and the study selection process is summarized in Figure 1. All included studies and individual case reports are listed and cited in Appendix 1.

Data Analysis

Two investigators independently reviewed all included articles and extracted data using a standardized collection form. Extracted variables included patient demographics (age and sex), anatomic location of the lesion (reported both by specific site and by organ system), tumor size (maximum width and length in centimeters), presenting complaint, symptom duration (reported in months or years), presence of pain or associated symptoms, descriptive features of the lesion (e.g., swelling, erythema, pruritus), disease progression timeline, malignant versus benign classification, and additional reported clinical or pathologic information. Discrepancies in data extraction were resolved through discussion and consensus. 

Descriptive statistics were used to summarize study variables. Normality of continuous variables was assessed using the Shapiro-Wilk test. Tumor size and disease duration were found to violate normality assumptions (Shapiro-Wilk p < 0.05) and were therefore analyzed using nonparametric comparisons between benign and malignant cases with the Mann-Whitney U test. Independent-samples t-tests were used only for variables that met normality assumptions. Statistical analyses were conducted using R version 4.5.1 (R Foundation for Statistical Computing, Vienna, Austria). All statistical analyses were exploratory and hypothesis-generating in nature; given the reliance on heterogeneous case-report data, no causal inferences were made.

Quality Assessment and Risk of Bias

The JBI Critical Appraisal Checklist for Case Reports was used as a guiding framework to inform data extraction and assess the completeness and clarity of reporting across included case reports [7]. Case reports were not excluded solely based on missing individual data elements, given the rarity of CCH and the descriptive aim of this review. As such, reports with incomplete information were included when sufficient clinicopathologic detail was available to confirm the diagnosis. Variables not reported in individual cases were recorded as ‘not listed’ in the dataset. 

Potential sources of bias include publication bias, reporting bias, selection bias related to the use of a single database (PubMed) and English-language restriction, time-lag bias, citation bias, and biases inherent to retrospective case report data. These limitations should be considered when interpreting the findings.

Results

Overall demographic and clinical characteristics of included cases are presented in Table 1, with individual case-level data provided in Appendix 1. 

**Table 1:** Summary characteristics of included clear cell hidradenoma cases (n=241) Anatomic locations are reported as described in the original case reports. Percentages are calculated using the total number of cases (n=241). Histologic classification was not reported in a substantial proportion of cases. SD: standard deviation

Variable	Overall
Total cases	241
Mean age, years (± SD)	54.7 ± 21.1
Female sex, n (%)	131 (54.4%)
Malignant cases, n (%)	35 (14.5%)
Nonmalignant cases, n (%)	65 (27.0%)
Histology not reported, n (%)	141 (58.5%)
Mean tumor size, cm (± SD)	2.84 ± 2.62
Mean duration, years (± SD)	3.34 ± 4.41
Most common locations	Genitourinary, face, head

Demographic and clinical characteristics of included cases are summarized in Table 2. A total of 241 CCH cases were identified, including both malignant and nonmalignant tumors. The mean age at presentation was 54.7 ± 21.1 years. Females accounted for a slightly greater proportion of reported cases than males (131 (54.36%) vs. 110 (45.64%)); no inferential statistical testing was performed for sex differences in the overall cohort.

**Table 2:** Demographics and characteristics of the CCH cases Age was compared using an independent-samples t-test due to approximate normality. Tumor size and disease duration were compared using the Mann–Whitney U test due to non-normal distribution. CCH: clear cell hidradenoma; cm: centimeter; min: minimum; max: maximum; n: number of cases; SD: standard deviation

Gender	Number	Percentage
Male	110	45.64%
Female	131	54.36%
Total	241	
Age in years		
Mean ± SD	Mean: 54.7	SD: 21.1
Min and Max	Min: 0.58	Max: 94
Histology		
Malignant	35	14.52%
Nonmalignant	65	26.97%
Not reported	141	58.51%
Longest length of the CCH in cm		
Mean ± SD	Mean: 2.84	SD: 2.62
Minimum and Maximum	Min: 0.20	Max: 20
Number of years of having CCH		
Mean ± SD	Mean: 3.34	SD: 4.41
Min and Max	Min: 0.2	Max: 20
Location of cell: (n, %)		
Genitourinary	30	12.45%
Face	29	12.03%
Head	27	11.20%
Upper extremity	24	9.96%
Lower extremity	23	9.54%
Breast	20	8.30%
Foot	16	6.64%
Hand	13	5.39%
Shoulder	11	4.56%
Chest	10	4.15%
Back	9	3.73%
Ear	8	3.32%
Flank	3	1.24%
Neck	3	1.24%
Eye	6	2.49%
Abdomen	6	2.49%

Histologic classification was reported for 100 cases, of which 65 (65.0%) were nonmalignant, and 35 (35.0%) were malignant; the remaining 141 cases (58.5%) did not report malignancy status. The mean tumor size was 2.84 ± 2.62 cm, and the mean reported lesion duration prior to diagnosis was 3.34 ± 4.41 years. The most frequently reported anatomic locations were the genitourinary system (12.45%), face (12.03%), and head (11.20%), as categorized in the original case reports.

Age was compared between groups using an independent-samples t-test due to approximate normality. Tumor size was compared using the Mann-Whitney U test due to non-normal distribution. Table 3 contains the comparison data between malignant and nonmalignant CCH. The Shapiro-Wilk test results show the p-value is 0.0746 for age and <0.0001 for the length of the CCH. The Mann-Whitney U test was performed to compare the cell sizes between the cancer vs. non-cancer groups. Key findings include a statistical difference in the size of CCH and the length of time patients had a CCH when comparing malignant and nonmalignant CCH. Malignant CCH had a mean length of 4.43 cm, whereas nonmalignant CCH had a mean length of 2.42 cm (p<0.01). Patients who had a malignant CCH had the tumor for a mean of 5.65 years, whereas patients with a nonmalignant CCH had the tumor for a mean of 2.9 years (p<0.05). No statistical difference was found for age of patient, gender, or location of the CCH.

**Table 3:** Comparison of malignant and nonmalignant clear cell hidradenoma Age was compared using an independent-samples t-test due to approximate normality. Tumor size and disease duration were compared using the Mann–Whitney U test due to non-normal distribution. Inferential statistical testing was not performed for anatomic location due to small cell counts and violation of chi-square assumptions. * indicates p < 0.05. CCH: clear cell hidradenoma; cm: centimeter; Min: minimum; Max: maximum; n: number of cases; SD: standard deviation

Description	Malignant (n=36)	Nonmalignant (n=65)	p-value
Age in year			
Mean ± SD	55.7 ± 21.2	53.1 ± 20.2	0.55
Minimum	8	1.5	
Maximum	91	91	
Gender: n			
Male	14	34	0.24
Female	21	31	
Longest length of the CCH in cm			0.023*
Mean ± SD	4.43 ± 3.58	2.42 ± 2.03	
Minimum	0.6	0.2	
Maximum	13.5	9	
Number of years of having CCH			0.048*
Mean ± SD	5.65 ± 5.94	2.90 ± 3.35	
Minimum	0.02	0.04	
Maximum	20	14	
Location of cell: n			
Genitourinary	6	11	
Face	4	6	
Upper extremity	4	9	
Head	3	8	
Lower extremity	3	4	
Breast	3	9	
Hand	3	0	
Abdomen	3	0	
Foot	2	5	
Chest	1	2	
Flank	1	1	
Eye	1	1	
Shoulder	0	4	
Back	0	3	
Ear	0	3	

Discussion

CCH, also referred to as acrospiroma, is a typically benign, slow-growing cutaneous adnexal tumor of sweat gland origin, although malignant transformation has been reported [1]. This systematic review synthesizes published cases to characterize trends in anatomic distribution, clinical presentation, and malignant transformation of CCH. Our analysis demonstrates that malignant cases tend to present with larger tumor size and longer disease duration compared with benign cases, findings that may aid clinicians in risk stratification and diagnostic evaluation. Recognition of these patterns may help inform clinical suspicion and management of CCH. Importantly, no formal evidence-based clinical guideline currently exists for the diagnosis or management of CCH due to its rarity and the predominance of isolated case reports in the literature.

One clinical pattern identified in this review was patient age. The mean age of patients with CCH in the present review was 54.7 years, which is higher than the mean ages reported by Hernández-Pérez et al. (37.2 years) and Lee et al. (48.9 years) [6,8]. This difference may reflect variations in study periods, reporting practices, and access to dermatologic care rather than a true shift in disease epidemiology. Accordingly, these findings should be interpreted as differences in reported age at presentation rather than evidence of increased prevalence in older populations.

Another significant demographic variable is gender. Previous studies have reported that benign CCH occurs more frequently in women than in men, with an approximate female-to-male ratio of 1.7:1 [6]. In our systematic review, benign tumors were more commonly reported in women, with 131 female patients (54.4%) and 110 male patients (45.6%), consistent with prior literature. In contrast, no statistically significant difference in malignant CCH was observed between genders (odds ratio=1.65; 95% CI 0.72-3.79; p=0.24), consistent with previous reports [9]. Accordingly, while a female predominance is observed among benign cases, this study’s findings do not support a gender-based difference in malignant transformation.

This study highlights another important feature of tumor presentation: the size difference between malignant and benign CCH. To the authors’ knowledge, this relationship has not been systematically evaluated in prior reviews. Malignant CCH tumors were significantly larger at the time of diagnosis, with a mean length of 4.43 cm compared with 2.42 cm for nonmalignant lesions (difference ≈ 2.0 cm; p=0.023). This finding suggests that larger lesion size at presentation may be associated with a higher probability of malignancy and underscores the importance of careful clinical and histopathologic evaluation of larger CCHs.

In addition, these data showed that malignant CCH cases had a longer mean reported duration prior to diagnosis compared with nonmalignant cases (5.65 vs 2.90 years, difference 2.75 years; p=0.048). This finding indicates that malignant cases had been present for a longer period before diagnosis; however, this observation should be interpreted cautiously, as it may reflect delayed clinical presentation, recall bias, or differences in symptom recognition rather than a temporal progression from benign to malignant disease.

Histopathologic features that characterize malignant CCH include necrosis, infiltrative growth, extension into surrounding tissues, nuclear atypia, increased mitotic activity (≥4 mitoses per 10 high-power fields), and lymphovascular invasion [10].

Among the 237 CCH cases included in this review, 35 cases (14.8%) were classified as malignant at the time of diagnosis. CCH most commonly involves the head, trunk, and extremities, with additional cases reported on the face, shoulders, breasts, and genitourinary region. In the present dataset, the genitourinary region accounted for the largest proportion of malignant cases by absolute count; however, differences in malignant versus nonmalignant distribution by anatomic location did not reach statistical significance (p=0.19). Hernández-Pérez et al. similarly reported the head as the most common site of tumor occurrence, followed by the upper limbs, trunk, lower limbs, and neck [6]. Overall, the anatomic distribution observed in this review is consistent with prior literature, while location-specific associations with malignancy could not be definitively established.

CCH can affect individuals across a wide age range and may arise in diverse anatomic locations. Awareness of its variable clinical presentation across specialties may facilitate timely recognition and appropriate management. Although most cases of CCH are benign and effectively treated with local surgical excision, lesions with atypical features, unusual locations, or concerning clinical behavior may warrant input from additional specialties. Management typically involves primary care and dermatology, with surgical specialties engaged based on lesion location. Oncology consultation is generally reserved for malignant or highly suspicious cases. Familiarity with the clinical spectrum of CCH across relevant fields may aid in early diagnosis and appropriate referral for definitive treatment.

Further Research and Limitations

Future research should focus on larger observational studies, including prospective and retrospective cohorts, to further characterize factors associated with malignant CCH. Molecular and genetic investigations may improve understanding of the pathogenesis of malignant CCH and help refine diagnostic classification rather than guide therapeutic intervention. Prior studies have identified a recurrent TORC1-MAML2 gene fusion at t(11;19)(q21;p13) in CCH, supporting a shared molecular feature with other adnexal neoplasms [3].

Imaging studies have demonstrated heterogeneous features of CCH across ultrasonography, color Doppler, CT, and MRI, including variable echotexture, vascularity, attenuation, and signal intensity, highlighting the potential role of multimodal imaging in lesion characterization [11]. On a molecular level, immunohistochemical analyses have shown that clear cells consistently express epithelial membrane antigen and cytokeratins such as CK10, CK17, and CK18, aiding distinction from histologic mimickers [4]. Collectively, continued clinicopathologic, imaging, and molecular studies may improve diagnostic accuracy and understanding of this rare tumor.

This systematic review identifies recurring patterns and characteristics associated with malignant and nonmalignant CCH. Differences in tumor size, duration at presentation, and anatomic distribution were observed between benign and malignant cases; however, these findings should be interpreted within the constraints of retrospective case-report data. Additional studies are needed to better characterize factors associated with malignancy in CCH and to inform evidence-based clinical guidance.

This study has several important limitations. First, the available literature on CCH is limited and largely composed of retrospective case reports, so this data should be interpreted as hypothesis-generating rather than confirmatory. This review includes a large number of individual case reports, which are summarized in Appendix 1 and cited here for completeness [4,5,11-230]. In addition, a substantial proportion of included cases did not report histologic classification as malignant or benign, limiting the generalizability of malignancy-specific analyses. Specifically, although malignant tumors were larger on average, the substantial variability in tumor size indicates considerable overlap between malignant and nonmalignant cases, and this finding should be interpreted cautiously. The literature search was restricted to PubMed and English-language publications, introducing potential selection and language bias. Publication and reporting bias are also likely, as atypical or malignant cases may be preferentially reported. Many case reports lacked complete clinical or longitudinal data, resulting in missing variables recorded as “not listed,” which limited certain comparative analyses. Additionally, although some publications reported multiple cases, analyses were descriptive and did not adjust for potential clustering within reports or institutions. Finally, the retrospective nature and heterogeneity of case-report data, as well as small sample sizes within individual anatomic regions, limit statistical power and generalizability.

Despite these limitations, this review provides a contemporary synthesis of reported cases and highlights areas where standardized reporting and larger observational studies are needed to improve understanding of this rare tumor.

## Conclusions

CCH is a rare skin tumor most commonly found on the head, neck, extremities, and, in malignant cases, the genitourinary tract. Consistent with prior literature, our review found a higher frequency of benign CCH in women, although malignant lesions did not show a clear gender predilection. Tumor size and longer lesion duration were more frequently associated with malignant cases in the available reports, suggesting potential markers of increased risk. Definitive diagnosis, however, relies on surgical excision with histopathological evaluation, which remains the cornerstone for distinguishing benign from malignant forms and determining appropriate management. Continued research with more comprehensive reporting of clinical, demographic, and pathological features is needed to clarify prognostic factors and improve the recognition and care of patients with this uncommon tumor.

## Appendices

Appendix 1 

**Table 4:** Case-level characteristics of clear cell hidradenoma included in the systematic review Case-level data from all included reports of clear cell hidradenoma, including demographics, tumor characteristics, anatomic location, and histologic classification when available. Missing data are left blank. The table is provided as supplementary material due to its size. References corresponding to the individual case reports summarized in this table are cited here for completeness [4,5,11–230]. CCH: clear cell hidradenoma; cm: centimeter; M: male; F: female

Author and Year of the publication	Gender	Age (year)	Location of the cell	Location by system	Size (width x length) in cm	Presenting Complaint	Number of months or years of having it	Painful or symptomatic (Y/N)	Description of the site (swollen, red, itchy, etc.)	Progression timeline	Cancerous (Y/N)	Additional Information
Kramer, 2022	M	60	Abdomen	Abdomen	1.5long	Not Listed.		Not Listed.	Not Listed.	Not Listed.		
Takada, 2021	M	19	Scapula	Upper back	2.5long	Skin tumor on the right scapular region, diagnosed as verruca vulgaris at another hospital.	3 years		Tissue damage is likely caused by repeated liquid nitrogen therapy.	Diagnosed as verruca vulgaris, tried liquid nitrogen therapy (not successful), kept increasing in size, another doctor diagnosed it as a keloid and tried triamcinolone acetonide injection (not successful).	The scapular region is acne-prone. The tumor presented as a lesion surrounded by acne, so it was initially thought to be a keloid. Preference for the scapular region may be due to strong cyclic tension due to postural ch	
Todi, 2021	F	32	Scalp	Head	9x6	Asymptomatic left-sided chronic scalp swelling over 4 years.	4 years		Scalp swelling.	Scalp swelling for over 4 years, no other symptoms.	N	First to report eccrine acrospiroma (hidradenoma) of such a large size on the scalp.
Wollina, 2021	M	53	Ankle	Lower extremity	Not Listed.		Not Listed.	This is the second reported case in the English literature.	N	Ankle - presentation at this location is extremely uncommon. It is difficult to differentiate nodular hidradenomas from basal cell carcinoma and melanoma.		
Park, 2021	F	42	Lower lip	Face	0.6x0.4x0.4	Enlarged cyst.	2.5 months	N	Not Listed.	Gradual enlargement of cyst over 2.5 months.	N	First reported case of benign hidradenoma of the lower lip.
Li, 2021	M	67	Scrotum	Genitourinary	3.2x2.3	Solitary scrotal mass, mild tenderness.	6 years		Not Listed.	Small nodule that increased in size over the past 6 years.	N	First report of giant vascular eccrine spiradenoma in the scrotal region.
See, 2021	F	20	Liver, chest wall	Abdomen	1	Chest wall nodule.	8 years		Not Listed.	Over 8 years: pulmonary cyst and liver mass removed but recurred.	Y	First instance of hidradenocarcinoma described in a patient with DICER1 syndrome, especially rare in young age.
Kundu, 2021	F	46	Leg	Lower extremity	13.5x7	Small nodule on left leg.	7 years		Sudden increase in size.	Left leg nodule of 7 years, rapid increase in size over 6 months, as well as pain.	Y	Malignant eccrine spiradenoma that arose from a benign lesion. Also found to have both carcinomatous and sarcomatous differentiation, which is rare.
Xing, 2021	F	53	Abdomen	Abdomen	3x2.5	Painful abdominal nodule.	1 year		Not Listed.	A painful nodule on the right abdominal wall was found 1 year ago. Recently increased in size, the patient's pain worsened.	The most common mode of presentation of eccrine spiradenoma is as a painful nodule. Pain due to nerves and capillaries surrounding the connective tissue capsule. Depicts ultrasound findings useful for diagnosis.	
Salim, 2021	F	35	Thigh	Pelvis	2x1	Thigh swelling for 1 year.	1 year		Not Listed.	Thigh swelling for 1 year, unchanged in appearance. Experienced increasing discomfort in the lesion with walking as the thighs rubbed together.	Mass was soft and fleshy, with a narrow stalk, resembling a papilloma. Unusual location on the thigh, had a papilloma-like configuration rather than a nodular configuration.	
Moore, 2021	F	19	Armpit	Upper extremity	Mass in left axillary.	7 years	N	Bleeding mass and upper extremity swelling.	Over 6 years, tumor progressed, recurred twice, even after removal.	Y	2 local recurrences of “atypical hidradenoma” developed into a hidradenocarcinoma metastasized to the liver, left adrenal gland, spleen, lungs, and thoracic vertebral body. First reported metastatic a	
Obermann, 2021	F	71	Flank	Torso	3x3	Rapidly enlarging nodule.	3 months	N	Not Listed.	Rapid enlargement over 3 months.	Y	First report of successful treatment with PD-1 immune checkpoint inhibition for hidradenocarcinoma. Cutaneous hidradenocarcinoma is extremely rare.
Gonzalez-Lopez, 2021	2M, 1F	29, 32, 51	Groin	Genitourinary	Not Listed.		Occasional decapitation secretions.	Not Listed.	N	All cases of CCH-like neoplasms with lymph node involvement reported so far involved a single lymph node in the axillary or inguinal regions and turned out to be benign. This study had a short follow-up		
Tran, 2021	1M, 1F	74, 39	Scalp, Flank	Torso		Not Listed.		Not Listed.	Not Listed.	2 cases are presented in a review of findings by MacKinnon, 2020. The author posits that apocrine hidradenomas and adenomyoepitheliomas can be differentiated histologically.		
Matsumoto, 2021	F	71	Breast	Chest	1.1x1x1	Breast mass detected on screening mammography.	N	Not Listed.	Not Listed.	N	The author illustrates the challenges with the diagnosis of breast spiradenoma, as it mimics adenoid cystic carcinoma.	
Al-Adawi, 2020	F	36	Breast	Chest	2x2	Painless lump in left breast.	4 months	N	Not Listed.	4 months of a painless lump in the left breast, seen on exam and ultrasound as a complex cystic mass with a solid vascular component.	N	First case to diagnose CCH of the breast via core needle biopsy, sparing the patient from wide local excision or mastectomy. CCH occurs more commonly in the left breast and presents as a painless mass.
Held, 2020	F, F	32, 75				Gluteal mass.		Not Listed.	3 years, unknown.	Y	2 spiradenocarcinoma cases are reviewed showing sarcomatous differentiation, both developed from a pre-existing, sporadic spiradenoma.	
Efremov, 2020	M	83	Hand	Upper extremity	1.2	Small mass on palmar surface of left hand.	5 years		Not Listed.	Mass had been slowly increasing in size over past 5 years and causing discomfort during daily activities.	Not very common location; this is the second report of CCH on palmar surface of hand.	
Hartford, 2021	F	42	Eyelid	Face	1.1x0.9	Painful lower right eyelid.	10 months	Y	Bleeding for the past 3 months from the lesion.	Gradually increased in size over 10 months.	N	A rare case of eyelid nodular hidradenoma, progressing rapidly with ulceration and bleeding which initially suggested malignancy.
Hudacko, 2021	F	55	Perineum	Genitourinary	1.2x0.8x0.7	Perineal cyst.		Not Listed.	Not Listed.	N	CCH of the perineum was originally misdiagnosed as an invasive papillary urothelial carcinoma.	
Memon, 2021	F	20	Breast	Chest	3.3	Slowing growing Breast mass.	1 year		Not Listed.	Slow-growing left breast cystic mass for one year.	N	Author describes the difficulty of diagnosing CCH from low-grade MEC.
Piqué-Duran, 2021	M	91	Forehead	Head		Lesion on forehead.		Not Listed.	Not Listed.	Y	Rare case of clear cell hidradenoma–basal cell carcinoma collision.	
Ishikawa, 2021	F	80	Leg	Lower extremity	2.2x1.5x1.4	Skin tumor.	1 year		Not Listed.	Lesion grew slowly over 1 year.	Hidradenocarcinoma is a rare malignant tumor in a sweat gland.	
Couselo-Rodríguez, 2020	1M, 1F	55, 65	Shoulder, thigh	Upper and lower extremity	1.3,1.5	Pigmented dark brown lesions.	N	Not Listed.	Slow-growing lesions.	N	Authors describe 2 cases of pigmented eccrine poromas that mimic basal cell carcinomas.	
Sato, 2020	M	76	Leg	Lower extremity	3.3x2.7	Nodule on leg.	10 years	N	Dome-shaped, dark red nodule.	10 years.	N	The first case of spiradenocylindroma that developed on the leg, that showed characteristics of both and spiradenoma and cylindroma.
Catteau, 2020	F	54	Vulva	Genitourinary	3.5	Painless cystic mass on the right minor labia.	Not Listed.	Past history of invasive breast carcinoma treated with chemo, radiation, surgery.	Y	Vulva is rare location. Also first time that p53 mutation and absence of HPV were detected in this lesion, ruling out HPV but a rare finding outside of HPV.		
Hopkins, 2020	M	63	Leg	Lower extremity	Painful hematoma-like cyst.	Y	Not Listed.	Cyst was recurrent and painful.	Spiradenoma presented with adenoid cystic carcinoma-like features, making accurate diagnosis challenging.			
Tran, 2019	F	39	Abdomen	Abdomen	4.5x4.5x3	Slight discomfort upon lying directly on the mass.	4 years		Not Listed.	Slowly increasing mass over 4 years.	Y	The author illustrates the first case of a spiradenoma giving rise to a primary cutaneous adenomyoepithelioma with malignant features.
Zeng, 2020	M, M	87, 68	Left mandible, Right mandible	Face		Discomfort when eating, painful.	2 years, 2 weeks	Y	Not Listed.	2 weeks to 2 years.	N	Authors added 2 cases of the rare “TPH-like tumor of the mandible (TPHLTM)”, both misdiagnosed as malignancies, pre and post-operatively.
Sprinkle, 2019	M	55	Foot	Lower extremity	4x1	Foot lesion.	5 years		Not Listed.	Mildly painful and nodular lesion on forefoot. Lesion was gradually enlarging over past 5 years, with dramatic increase over last 6 months.	N	More commonly found in upper body, so unusual to see solid cystic hidradenoma on forefoot.
Macías Del Toro, 2019	F	89	Supraclavicular region	Shoulder		Not Listed.	20 years	N	Not Listed.	20 years progression of asymptomatic lesion. Stable over time.	Snow-falling sign in a long-standing tumor.	
Cukic, 2019	F	75	Left ear	Face	1.5	Left ear growth.	15 years	Y	No ulceration or bleeding.	Growth on left ear present for 15 years, which increased in size and became painful over 2 months.	Only case of eccrine spiradenoma described on the antitragus of the auricle.	
Ferdez, 2019	M	70	Chin	Face	3.5x3.5	Chin lesion.		Not Listed.	Enlarging mass on chin over 7-8 years. Shave biopsy revealed apocrine hidradenoma. Initially thought negative for malignancy but incisional biopsy showed malignant features.	Incisional biopsies are needed to capture as much of the tumor base as possible for hidradenoma, as a superficial biopsy may only capture exophytic granulation tissue.		
Metovic, 2019	F	84	Breast	Chest	2	Nipple lesion.		Not Listed.	Enlargement of right nipple with skin ulceration and eczematous changes, causing suspicion for Paget's disease.	First case of eccrine spiradenoma arising under nipple.		
Yilmaz, 2019	F	38	Left Breast	Chest	3x1.5	Palpable mass.	N	Not Listed.	Unknown.	N	This is the first case report to describe MRI findings of a breast hidradenoma.	
Jaitly, 2019	F	20	Breast	Chest	5x4	Breast mass.	1 year		Not Listed.	Right-sided breast mass has been present for 1 year. Recently increased and size, caused pain and serosanguinous discharge with overlying skin ulceration.	Nodular hidradenoma can mimic breast carcinoma, can be distinguished by superficial location immediately beneath the skin.	
Kanwaljeet, 2017	M	52	Chest	Chest	1.2x1	Painful swelling.	4 years	Y	Not Listed.	Painful swelling of the left-sided chest mass for 4 years. Mildly increased in size.	N	Malignant spiradenoma is rare and the rate of malignant transformation of this benign tumor is very low with a metastasis rate of 50% which can prove fatal for the patient.
Choi, 2018	F	66	Breast	Chest	2.8x1.9	Palpable breast lump.	2 years		Not Listed.	Slowly growing palpable lump in right lower inner breast over 2 years. Underwent FNA, lesion collapsed, 2 years later increased in size, resected.	Poroid hidradenoma, thought to have slow growth rate, not common in breast, may resemble intraductal papilloma.	
Yao, 2018	F	36	Leg	Lower extremity	1x1.5	Mass on leg.	2 years		Not Listed.	Right lower leg mass was stable for 2 years but enlarged during pregnancy.	Pregnancy can cause increase in eccrine activity, which likely contributed to growth of eccrine sweat gland tumor on leg. This is less common on lower extremities.	
Simões, 2018	M	26	Scrotum	Genitourinary	5	Mass on scrotum.	N	Not Listed.	Following excision of slow growing nodule, a fast-growing, painless left inguinal adenomegaly was resected.	Y	In Portuguese.	
García del Pozo, 2018	M	81	Knee	Lower extremity	0.6	Lesion on knee.	1 year		Not Listed.	Asymptomatic lesion growing in size over 1 year.	Pedunculated presentation of apocrine hidradenoma is rare. Resected with no recurrence after 1 year.	
Kane, 2017	M	85	Foot	Lower extremity	5.5x6.5x0.7	Sanguinous drainage form the lesion.	15 years	N	Not Listed.	Enlargement over a timeline of 15 years.	Y	Malignant hidradenocarcinomas are rare, especially in the lower extremity. In this case it was ulcerated.
Vázquez-Osorio, 2017	F	87	Back	Back	1.85x1.23	Painful lesion on lower back.	Y	Dimple-shaped lesion.	Patient could not remember when it first appeared.	NH usually affects females in their 40s and 50s. Although a benign neoplasm, NH usually recurs after incomplete removal. Cutaneous ultra-sound can guide the presurgical diagnosis of NH.		
Patel, 2018	M	60	Knee	Lower extremity	Cystic lesion.	10 years		Not Listed.	Stable for 10 years but sudden growth over 3 months.	Y	Rare case of malignant hidradenocarcinoma arising from preexisting benign hidradenoma. These are very rare in the lower extremities.	
López Rojo, 2018	F	76	Breast	Chest	1.9	Palpable mass.	6 months	N	Not Listed.	Increase in size over 6 months.	Y	Initially diagnosed as benign CCH, clear-cell hidradenocarcinoma may start as slow growing but turn aggressive.
Mahipathy, 2017	F	45	Hand	Upper extremity	4x4x2.5	Hand swelling.	2 years	N	Swelling.	Noticed small painless swelling on dorsum of hand 2 years ago. Was near base of thumb but thumb extensors were normal.	Large lesions on hand, one of earlier reports of occurrence.	
Shalini, 2018	M	45	Thigh	Lower extremity	5x4	Slowing growing swelling over the thigh.	10 years	N	Swollen.	Slow increase in size over 10 years.	N	Author proposes using FNAC to differentiate chondroid syringoma mimicking a nodular hidradenoma and stresses the importance if using adequate cytological criteria to aid in differential diagnosis.
Hoashi, 2017	M	79	Arm	Upper extremity	1.5x2.0	Nodule on upper arm.	2 months		Pinkish nodule.	Development over 2 months.	Y	Malignant nodular hidradenoma can mask diagnosis of lung cancer and detailed lung evaluation with immunohistochemical profiling is recommended.
Park, 2017	F	61	Chin	Face	2.0x2.0x0.8	Swollen, leaking discharge from chin lesion.	8 months		Not Listed.	Slow growth over 6 months with serous discharge over past 2 months.	N	Although ulcerated lesions may suggest basal cell carcinoma, clear cell hidradenoma can be diagnosed by biopsy and histology.
Martinez-Cabriales, 2017	M	32	Scalp	Head	1.5x1	Lesion on scalp.	2 years		Bluish elevated nodule.	2 years of asymptomatic nodular lesion in left parietal region. Presented as smooth blue elevated nodule.	N	Hidradenocarcinoma looks clinically similar to hidradenoma, which frequently arises from the apocrine glands and not from the eccrine gland.
Chambers, 2017	F	58	Breast	Chest	1.5x1	Dx during well woman physical exam.	Not Listed.	Not Listed.	Y	Malignant clear cell hidradenoma is rare in the breast and tend to be aggressive. Radiologic and histologic techniques can help distinguish this malignancy from others.		
Diab, 2017	M	73	Groin	Genitourinary	Mass in left groin.	8 years		Not Listed.	Presented in 2009 with a nodular lesion on the dorsal aspect of the left great toe.	Y	First case of acral malignant acrospiroma with reported next-generation sequencing results. Malignant acrospiroma is a rare tumor that originates from the eccrine sweat glands and is aggressive in it	
Jacquemus, 2017	F	67	Arm	Upper extremity	Rapid growth of long-standing tumor.	Not Listed.	Rapid increase in size.	Y	Preexisting benign spiradenoma progressing to malignant eccrine spiradenoma.			
Roblez-Méndez, 2017	M	70	Arm	Upper extremity	Progressive enlargement of nodule.	Red, firm tumor.	Progressive increase in size over 7 months.	N	Dermoscopy alone may not be enough; diagnosis requires the use of histopathology to differentiate from eccrine poroma or amelanotic melanoma.			
Reddy, 2017	M	70	Scalp	Head	0.5	Papule found during routine skin cancer screening.	Not Listed.	Multiple BCCs in the past.	N	Cutaneous oncocytic hidradenoma is very rare and can be confused with basal cell carcinoma.		
Au, 2017	F	36	Groin	Genitourinary	Subcutaneous mass in left groin.	8 years		Not Listed.	Recurrence after 8 years.	Y	Benign metastasis and tumor of uncertain malignant potential are terms that describe cases where lymphatic system is involved. Authors suggest the term “CCNH-like tumor of uncertain malignant potential	
Espejo Pérez, 2016	F	79	Arm	Upper extremity	Pedunculating nodule on left forearm.	Violet and firm.	Not Listed.	Nodular hydroadenoma can be confused with many other diseases, among which are dermatofibroma, papilloma, lipoma, hemangioma, blue nevus, epidermoid cyst, basal cell epithelioma and spinocellular epit				
Sharma, 2016	F	50	Breast	Chest	3.4x2.5x2.3	Lump in left breast.		Not Listed.	Not Listed.	N	Poroid hidradenoma in the breast is very rare. Its manifestation an be confused with eccrine spiradenoma, epidermal inclusion cyst, nodular hidradenoma, and a malignant breast carcinoma.	
Desai, 2016	M	67	Mandible	Face		Nodular growth.		Not Listed.	Not Listed.	N	Tubulopapillary hidradenoma is extremely rare in the mandible.	
Hsieh, 2016	F	50	Breast	Chest	3	Lump in Breast.	15 years		Not Listed.	Not Listed.	Y	Tumor thought to have originated from mammary glands, diagnosed as a clear cell variant mucoepidermoid carcinoma.
Bajaj, 2016	F	8	Scalp	Head	13x10	Lesion on scalp.	3 months		Not Listed.	Painless scalp swelling for 3 months, resected, recurred and resected 11 months later. Presented a year later with another recurrence, excised, recurred and excised again. Excision showed infiltrating tumor tissue to periosteum, vascular invasion, diagnosis of malignant hidradenoma. Recurrence again, showing metastases to lungs, spine, sacrum, humerii. Chemo and palliative care.	Y	Known to occur in elderly, this is a rare case of metastasizing malignant hidradenoma in a child.
Bhullar, 2016	M	13	Arm	Upper extremity	Slow-growing painless nodule.	18 months	Site of BCG scar.	Painless fungating nodule developing over 18 months.	N	Rare occurrence of benign painless erythematous fungating nodule on the left upper arm at the site of the BCG vaccination.		
de Meo, 2016	F	70	Scapula	Shoulder	1x0.8	Mildly tender nodule.	7 months	N	Red, dome-shaped, tender.	Not Listed.	N	Apocrine hidradenoma can be difficult to differentiate from benign mimicking lesions and can be diagnosed by histopathological analysis.
Nakazato, 2016	F	67	Ear	Face	3.5x3.0x2.5	Tumor.	Unknown due to patient's dementia	Reddish.	Not Listed.	N	This is a case of nodular hidradenoma of the auricle mimicking squamous cell carcinoma.	
Elbendary, 2016	F	39	Vulva	Genitourinary	2x3	2 labial masses.		Cloudy white exudate from larger mass.	Not Listed.	N	Case documents hidradenoma papilliferum and demonstrates the benign nature of oncocytic metaplasia in it.	
Tingaud, 2016	M	49	Groin	Genitourinary	Lymph node enlargement.	6 years	N	Not Listed.	Stable inguinal lymph node enlargement for 6 years.	N	The author illustrates the difficulty of diagnosing primary nodal hidradenoma, “benign metastasis” of a cutaneous hidradenoma and hidradenocarcinoma.	
Rooper, 2016	F	59	Breast	Chest		Superficial Breast mass.	Not Listed.	Not Listed.	N	Although FNAC suggested primary mammary carcinoma, histological findings showed atypical hidradenoma.		
Namiki, 2016	M	79	Face	Face	1.6x1.4	Tumor below right nostril.	N	Dome-shaped, dark brown to black tumor.	Gradual enlargement of plaque over several years.	Y	Basal cell carcinoma with myoepithelial differentiation presented as a dome-shaped tumor, other possibilities ruled out by histological evidence.	
Jones, 2016	M	67	Forehead	Face	1.1x1.1	Tender nodule undergoing transient enlargement.	2 years		Hyperpigmented nodule.	Over 2 years, nodule became inflamed twice, growing transiently on each occasion.	Spiradenocylindroma are difficult to differentiate. Two distinct histomorphology patterns are evidenced, suggesting presence of both spiradenoma and cylindroma. The lesion was excised.	
Jinnah, 2016	F	56	Palm	Upper extremity	3.8x3.4x2.5	Mass on right palm	1 year	Y	Bluish discoloration.	Gradual enlargement over 6-12 months.	Y	Although usually found in the head and neck region, a hidradenocarcinoma found as soft tissue mass on the palm with FNA and resected.
Huddleston, 2016	M	70	Anogenital area	Genitourinary	0.2	Hemorrhoids, perianal itching, pain with bowel movements, occasional bleeding, and near complete fecal incontinence.	Y	Cuts in anoderm and perirectal skin.	Not Listed.	N	Hidradenoma papilliferum in men is rare. Differential diagnosis was aided by histopathological evidence to rule out thrombosed external hemorrhoids, anal abscess, viral warts, and epidermoid carcinoma	
Tarantino, 2016	M	81	Chest	Chest		Chronic, bleeding and painful lesion.	10 years	Y	Unspecified nodule with two superimposed nodules.	Occasional bleeding and serous fluid leaking over 10 years.	Lesion would bleed and leak serous fluid over 10 years. Diagnosis of nodular hidradenoma was made.	
Horie, 2016	F	69	Flank	Torso	0.6diameter	Mild nodular pain.	2 months	Y	Red hemispherical nodule.	Slow progression over 2 months.	N	Nodular hidradenoma diagnosis using ultrasonography.
Myreddy, 2015	M	45	Ear	Face	4x3	Mass over right ear lobule.	Ulcerated bleeding mass.	Mass present over 6 months with dull aching pain. Tender to deep palpation, firm. Patient felt abnormal fullness and ear discomfort. Biopsy showed clear cell hidradenoma.	Skin adnexal tumor in ear lobule is rare.			
Jagannatha, 2016	M	76	Scalp	Head		Large, recurring, fungating scalp swelling with focal seizure and drowsiness.	14 years		Not Listed.	Progression over 14 years resulting in a "moth-eaten" appearance of the parietal bone which was lytic.	Y	Extremely rare case of brain invasion of hidradenocarcinoma. Wide excision of the lesion following a diagnosis of histopathology-evidenced hidradenocarcinoma. No recurrence after 2 years.
Vasconcelos, 2015	F	40	Breast	Chest	5	Exudative nodule on the breast.	5 years	N	Crusty nodule with exudate.	Slow growing mass over 5 years.	N	Benign solid-cystic hidradenoma could potentially be confused with breast carcinoma using FNA and biopsies.
Chan, 2016	M	70	Finger	Upper extremity	Arthritic change.		Not Listed.	Not Listed.	Rare hidradenocarcinoma of the finger mimicking a giant cell tumor.			
Imafuku, 2016	M	81	Ankle	Lower extremity	0.5x1	Lesion with fluid secretion.	5 years	Y	Dark blue/purple, round nodule with whitish exudate.	Growth over 5 years from a tiny papule.	N	Solid-cystic hidradenoma is suggested as a differential diagnosis where viscous fluid secretions are present.
Luzar, 2015	F, F	19, 53	Back, arm		1	Painful, slightly raised lesions.	Y	Not Listed.	10 years, unknown.	N	Predomitly intraneural growth of a spiradenoma and a hidradenoma are reported to not signify maligcy.	
Plantier, 2015	F	70	Scalp	Head	2.5	Growing lesion.	4 years		Pink, whitish, shiny plaque.	Growth over 4 years.	N	History of patient warranted differential diagnosis of carcinoma but histological evidence pointed to a clear cell hidradenoma.
Patel, 2015	F	73	Hand	Upper extremity	2x1.5	Hypertensive urgency and neck pain.	Not Listed.	Unusual growth seen on left hand, present for over a year. Difficult to obtain radiography, US and MRI due to contracture of hand. Large incisional biopsy performed and revealed nodular hidradenoma.	Not Listed.			
Pandey 2015	M	50	Axilla	Upper extremity	5x4	Axilla swelling.		Swelling of axilla. Overlying skin was indurated and inflamed.	Left axilla swelling for 2 months. Was initially small then grew. Patient was also febrile off and on over 2 months. Thought to be soft tissue sarcoma, then fine needle aspiration revealed malignant eccrine acrospiroma.	Malignant eccrine acrospiroma is hard to correctly diagnose preoperatively. Biopsy and histology required to accurate diagnose. Misdiagnosis may be due to rarity of neoplasm and failure to identify mo		
Brodbeck, 2015	F	44	Thumb	Upper extremity	Tumor.			Not Listed.	Not Listed.	Y	Rare aggressive tumor digital papillary adenocarcinoma was excised in 2 operations post diagnosis, thumb reconstructed and amputation was avoided.	
Nguyen, 2015	F	26	Toe	Lower extremity	2	Toe mass.			Not Listed.	Toe mass resected, originally though to be digital papillary adenocarcinoma but later confirmed as benign poroid/clear cell hidradenoma.	Poroid hidradenoma is a rare tumor with characteristics of both poroma and hidradenoma. Atypical pathology seen in this case. May present similarly to clear cell hidradenoma. First case of benign nodu	
Egesi, 2014	F	70	Scalp	Head	2.5	Nodule.	2 months	N	Firm, red nodule.	Rapid enlargement over 2 years.	N	History of breast carcinoma in the past 2 years. Rapid growth of a red nodular tumor indicated malignancy but turned out to be CCH.
Sorin, 2016	M	75	Scalp	Head		Multiple subcutaneous blue and purple nodules.	1 year		Blue and purple nodules on the scalp.	Nodules appeared gradually over 1 year.	N	Zosteriform configuration of multiple eccrine spiradenomas of the scalp are extremely rare. Non-malignant nodular adnexal tumor was removed.
Boeßert, 2015	M	52	Ear	Face	1x0.5	Recurrent colds.		Redness surrounding the ear canal.	For several months, patient reported a feeling of fullness and a new, low-grade hearing loss on the left.	N	Ear microscopy revealed a cystic-blistered tumor permeated with increased vessels and raspberry-like tumor, which seemed to emanate from the anterior wall of the ear canal in the area of the tympanome	
Uke 2010	Female	44	Labia majora	Genitourinary	Small, exophytic growth on the labia majora.	14 years		Not Listed.	Not Listed.	Y	Initially reported as a maligt tumor. Repeat vulvar smear shows groups and clusters of benign-looking glandular cells and interpreted as a benign tumor. Histology- hidradenoma papilliferum.	
Montenegro 2010	Female	75	Perianal	Genitourinary	1	Not Listed.		Not Listed.	Not Listed.	Eccrine acrospiroma. Patient underwent adjuvant chemotherapy in the Oncology Service and died 1 month later.		
Vazmitel 2009	Female	41	Vulva	Genitourinary	2	Nodular asymptomatic lesion on the vulva.	Not Listed.	Not Listed.	Disease-free 8 months.			
Demellawy, 2008	F	46	Vulva	Genitourinary	1.0x1.0	Persisting rubbery painless subcutaneous nodule.	"several months"	N	Single rubbery subcutaneous nodule.	Several months with slow progression; removed with simple excision; 8 months follow-up and no recurrence.	N	First reported case in this location.
Iannicelli, 2008	F	56	Perineal	Genitourinary	"large"	Rectoragia and pain in inferior abdomen; perineal lesion.	2 years	Y	Perineal and perianal ulcerated lesion extending along the medial portion of the thigh. At the rectal and gynecological examination, the anal canal and the posterior and lateral wall of the vagina, beginning from the perianal margin, appeared to be involved.	Diagnosed and had perianal eccrine poroma removed 10 years prior; 8 years went by well, then presented with inferior abdominal pain and rectoragia; Mild abdomino-perineal resection with colostomy plus total hysterosalpingooophorectomy en bloc and an extended colpo-vulvectomy with a cutaneous wide excision of the tumor; 13 months follow-up no recurrence.	Y	First reported case to have studied a porocarinoma with MRI.
Lan, 2003	F	81	Face	Head		Lesions on left side of face.	1 week		Discrete with smooth surface lesions that coalesced into large plaques.	2 years prior, she had a surgical excision of a cutaneous horn with the pathological diagnosis of Bowen's disease; patient comes in with multiple erythematous indurated papules over the left side of face; patient refused treatment.	Y	Abnormal expression of interleukin-2 receptors after stimulation.
Kose, 2006	F	50	Gluteal region	5.5x5.0	Enlarging, swelling following an intramuscular injection.	1 year	N	Reddish-blue cystic mass noted on the left gluteal region.	Not Listed.	Y	According to the history obtained from patient, the swelling began to appear following an intramuscular injection. Diagnosis of eccrine acrospiroma.	
Punia, 2001	F	34	Forearm		3.5x2.5x1.5	Small swelling with history of discharge from the swelling.	18 years	Y	2.5 X 2.5 cm irregular, firm swelling with opening on surface; fixed to overlying skin.	Not Listed.	Diagnosed as eccrine acrospiroma.	
Long, 1998	F	66	Sternum		2.5x2x2.2	On admission patient presented with ulcerative mass located at base of neck over the sternum.	10 years	Y	Grossly the tumor invaded the skin, sternum and surrounding soft tissue. The tumor itself was tan and rubbery.	10 years.		Diagnosis of malignant eccrine acrospiroma.
Kiehl, 1997	F	72	Back		0.8x0.8x0.4	Local recurrence following incomplete excision. Originally came in to get rid of a lesion thought to be Basal Cell Carcinoma.	10 years, excised and grew back in 6 months	pink, nodular, painless growth.	Not Listed.	Incomplete excision caused regrowth of the tumor.		
Kumar, 1996	F	75	Breast		2x3	Breast lump for 6 weeks with ulceration of overlying skin.	6 weeks	Y	well-circumscribed, firm, nontender, discrete, mobile, ulceration of skin over it.	6 weeks.		Due to lack of cytological information on clear cell hidradenoma, this case was wrongly diagnosed as breast carcinoma initially when FNA was done.
Cyrlak, 1995	F	25	Right breast	5x7x6	Mass and a clear yellow discharge from the right nipple.	1 year	Y	Mass protruded substantially above the surface of adjacent breast tissue. Was well-encapsulated and was associated with superficial skin discoloration.	Slow growing over 1 year.	Y	Patient had delivered her 4th child 6 weeks prior to presenting in clinic. Pregcy did not accelerate the growth of the mass. Patient had ceased breastfeeding at the time of presentation.	
Cohen, 1992	F	36	Scalp		5mmnodule	Papule found by hairdresser.	4 months	N	Flesh colored papillary surface.	Enlarging and would bleed when traumatized.	Not Listed.	
Nasit, 2014	Male	70	Scalp	Head	3x2.5x2	Slowly growing nodule over scalp x 10 years. Pain, serous discharge, and ulceration of skin x 1 month.	10 years	Y	Solitary, non-tender, firm to hard, skin colored swelling 3 x 2.5 x 2 cm over fronto-parietal region of scalp.	Not Listed.	Histology- well-circumscribed, encapsulated solid-cystic lobulated intradermal tumor made of 2 cell types: eosinophilic and clear cells. No atypia, invasion, necrosis, and mitosis seen. Disease-free 1	
Brasileiro 2014	Male	49	Chest	Chest	3diameter	Asymptomatic, ulcerated, pink exophytic nodule.	13 months	N	Ulcerated, pink exophytic nodule measuring 3 cm in diameter.	Similar lesion 8 months prior, excised at another center, no histology results from that time, rapid relapse and growth of lesion after procedure.	Histology- poorly circumscribed ulcerated tumor in continuity with epidermis and part of hypodermis; several areas of necrosis and mitoses; +AE1/AE3, -CEA/EMA. Negative sentinel LN biopsy. Disease-fre	
Aung, 2015	Female	65	Axilla	Upper extremity	0.6x0.5	Asymptomatic, protuberant pink nodule on right inferior axilla x 1 week.	1 week	N	Firm solitary smooth-surface nontender pink nodule, 0.6 x 0.5 cm; gray-white lobulated to slightly cystic nodule adjacent to skin surface.	Not Listed.	Histology- circumscribed, nonencapsulated bland dermal tumor with poroid and clear cells and foci of ductal differentiation; adjacent short, curled frayed basophilic thickened elastic fibers in reticu.	
Lee, 2014	Male	7 months	Wrist	Upper extremity	1x1	Dome-shaped, erythematous to purplish, firm nodule on flexor side of right wrist.	5 months		Not Listed.	Slowly growing from 3 month old to 7 month old.	Histology- nodular, solid cystic lesion in the dermis compose of basaloid eosinophilic cells and clear cells with duct-like structures in center of solid lesion. Spontaneous regression after 3 months.	
Sbai, 2014	Male	60	Scalp	Head	4	Gradually increase in size nodule on scalp.	1 year		Not Listed.	Not Listed.	Histology- maligt adnexal tumor of apocrine hidradenocarcinoma type, absent tumor residue. Tx with monitor. Recurred in 39 months.	
Bae, 2014	Female	89	Ear	Head	4x3.5	Hard, nontender solitary mass, insidious in onset, gradually increased in size.	2 years		Not Listed.	Not Listed.	Histology- clear cell hidradenocarcinoma - solid, necrotic lesion composed of infiltrating atypical cells with large polygonal nucleus and eosinophilic or clear cytoplasm. No evidence of local recurrence.	
Sehgal, 2014	Female	30	Breast	Chest	3x2.5	Not Listed.	10 months	N	Not Listed.	Not Listed.	Histology- clear cell hidradenoma. Disease free 10 months.	
Morcos, 2014	Male	55	Eye	Face		Pigmented elevated lesion of right eye since 12 years old, growing for the past 2 years.	2 years		Not Listed.	Not Listed.	Tumor: + Melan A, S100 protein, HMB45. Dx: balloon cell nevus.	
Jakobiec, 2014	Female	46	Eye	Face		Recurring papilloma of L medial upper eyelid margin, 5 mm diameter cutaneous mass thought to be cystic, beneath papillary lesion.	Not Listed.	Not Listed.	Histology- epidermal keratinizing squamous papilloma surmounting a circumscribed dermal papillary hidradenoma composed of deeply eosinophilic columnar cells, intraductal proliferation of tumor extending.			
Kurashige, 2014	Female	43	Vulva	Genitourinary	Skin-colored cystic nodule.	Not Listed.	Not Listed.	Histology- neoplasm formed by tubular structures consisting of 2 types of pleomorphic cells, columnar cells in luminal layer and cuboidal cells in basal layer; wide divergent lobular ductal structure.				
Shahmoradi, 2013	Male	31	Head	Head	2x2.5	Asymptomatic, cone-shaped solitary nodule on occipital region 2 years ago, started to grow quickly 4 months ago.	2 years	N	Not Listed.	Not Listed.	Histology- tumor lobules composed of cell masses separated by eosinophilic, homogenous material, with 2 types of cells - one polyhedral with rounded nucleus and slightly basophilic cytoplasm; another	
Mitamura, 2014	Female	46	Chest	Chest	5diameter	Nodule on the left chest.	5 years	N	Dome-shaped, elastic, hard, red-to-purple hemorrhagic nodule.	Not Listed.	Histology- atypical tumor cells with hyperchromatism and pale cytoplasm stained +PAS. Etc.	
Ramli, 2014	Female	65	Vulva	Genitourinary	1diameter	Painless vulvar nodule x 8 months.	8 months	N	Mobile, pink.	Not Listed.	Histology- papilliferous hidradenoma. Disease-free for 2 years.	
Rahim, 2013	Female	57	Neck	Neck		Tender enlarging lesion on anterior neck x 5 months.	5 months (enlarging)	Not Listed.	Excision of lesion at this site 14 years before.	Not Listed.		
Arsalan-Werner, 2013	Female	73	Wrist	Upper extremity	Not Listed.		Not Listed.	Not Listed.	Not Listed.			
Cauthon, 2013	Female	64	Foot	Lower extremity	0.7x0.7x0.9	Lump on left heel that has been getting bigger, nickel-size.	Not Listed.	Initially dx lipoma vs. ganglion cyst.	Histology- eccrine hidradenoma, infiltrative growth pattern, populations of polyhedral cells with basophilic cytoplasm, rounded nuclei, and pronounced nucleoli; numerous cells with glycogen-rich clear			
Paranjyothi, 2013	Female	67	Face	Face	2x2	Asymptomatic swelling of left cheek.	5 months	N	Not Listed.	Initially dx lipoma vs. fibroma or cysticercus cellulose.	Cystic lesion, filled with yellow, viscous, odorless fluid with white flecks. Histology-epidermoid cells on background of eosinophilic material similar to keratin.	
Christakopoulos, 2014	Male	84	Eye	Face		Insufficient eyelid closure.	Not Listed.	Not Listed.	Histology- epithelial cells with intervening strands of hyalinized stroma , +CK-KAM 5.2, BER-EP4, p63, smooth muscle actin-+.			
Panasiti, 2013	Male	62	Scalp	Head	0.5	Asymptomatic lesion of the scalp, blue-colored.	7 months	N	Not Listed.	Not Listed.	Histology- intradermal neoplasm with complex pattern of tubules connected in a labyrinthine manner, with several papillary folds projecting into a cystic lumen without epidermal connection.	
Konstantinova, 2014	Female	63	Vulva	Genitourinary	1.0x0.5	Not Listed.		Not Listed.	Not Listed.	Histology- similar to a mammary fibroadenoma.		
Nazerali, 2013	Male	87	Left finger	Upper extremity	1-1.5diameter	Asymptomatic left ring finger mass.	3 years	N	Not Listed.	Recurrence after liquid nitrogen cryotherapy.	Not Listed.	
Drvis, 2012	Female	78	Ear	Head	6diameter	Pain and fullness with permanent otorrhea from the right ear.	1 year	Y	Not Listed.	Not Listed.	Disease-free for 1 year.	
Katz, 2013	Female	63	Eye	Head		L upper eyelid fullness.	18 months	Not Listed.	Not Listed.	Not Listed.		
Hama, 2013	Female	37	Perianal	Genitourinary	0.7	Asymptomatic papule in perianal area.	1 month	N	Ulcerated.	Not Listed.	Histology- dermal nodule well circumscribed by fibrous capsule in middle and lower lesion but not in the upper lesion, cystic branching spaces.	
Takasaka, 2014	Male	48	Leg	Lower extremity	2.5x2.0	Not Listed.	2 years		Skin-colored, elastic, soft, subcutaneous nodule.	Not Listed.	Histology- excised nodule showing cystic structure mixed with solid portion protruding from cyst wall. Solid portion composed of fibrous tissue at base and poroid cells and cuticular cells forming lum	
Jain, 2012	Male	80	Left eyelid	Face	1.2(diam)	Cystic swelling of the left lower eyelid that has been gradually increasing in size.	Pearly, firm, cystic reddish tumor nodule at the lower eyelid margin, thickened and indurated.	Not Listed.	Not Listed.			
Wenzel, 2012	Male	86	Foot	Lower extremity	Cellulitis, edema, and abscess formation on distal left 5th digit.	Not Listed.	Presented as cellulitis.	Histo: asymmetrical neoplasm of skin, adnexal in origin, composed of lobules and nests showing cyst formation.				
Benmously-Mlika, 2011	Female	19	Scapula	Back		Not Listed.		Not Listed.	Not Listed.	Not Listed.		
Abudu, 2011	Female	70	Breast	Chest	20.0x16.0	A slowly growing painful, palpable mass of the left breast.	5 years	Y	Nodular, cystic, non-tender, freely mobile; overlying skin with cutaneous veins, bloody nipple discharge.	Not Listed.	Histology- well-encapsulated tumor composed of arborizing papillary glandular pattern; lined by pseudostratified, bland-looking epithelial cells, and possessed a loose fibrous connective fibrovascular stroma.	
Fernandez-Flores, 2012	Male	38	Perianal area	Genitourinary	1.1diameter	Nodule on the perianal area.	"Several months"	Skin-colored tumor.	Not Listed.	Diagnosed with pseudo carcinomatous hyperplasia.		
Jakate, 2012	Male	62	Occiput	Head	2diameter	Cystic mass of the occiput.	2 years		Not Listed.	Not Listed.	Histology- partially dermal solid and cystic proliferation, with areas of glandular differentiation with cuboidal to columnar cells lining luminal and cystic spaces. +S100, cytokeratin, smooth muscle.	
Demirci, 2011	Female	45	Abdomen	Abdomen	6.5x5x4	Small nodular asymptomatic lesion with central ulceration and minimal hemorrhagic discharge on her anterior abdominal wall.	4 years	N	Soft, reddish purple nodule with lobular appearance and ulceration.	Not Listed.	Histology-biphasic smaller dark polygonal cells and larger clear cells and coarse nuclear chromatin; + duct like structures.	
Lerner, 2011	Male	73	Scalp	Head		Nodule.	"Recent onset"	Not Listed.	Initial nodule. Follow up 2 months after initial presentation - development of several 2cm nodules at the superoposterior resection margin. Within weeks, developed multiple, rapidly growing nodules, all within same locoregional area, as well as palpable ipsilateral level 2 and 3 cervical lymphadenopathy. 3 months after treatment with oral capecitabine, resolved.	Histology- malignant hidradenocarcinoma. +pankeratin, cytokeratin 7, and epithelial membrane antigen.		
Torrado, 2011	Female	84	Eyelid	Face		Not Listed.	N	Firm, non-infiltrating bilobular mass involving both puncta and induced mechanical ptosis.	Not Listed.	Not Listed.		
Singhal, 2010	Male	35	Shoulder	Upper extremity	Painful restriction of right shoulder motion with extreme motions of the joint, more so in activities involved raising the arm in an overhead position.	3 months	Y	Not Listed.	Small nodule observed 20 years back, increased in size gradually until development of restriction of shoulder movements.	Histology- well-circumscribed, fibrous capsule, glandular or cystic spaces; cuboidal cells and papillary structures; contained eosinophilic granular material; many psammoma bodies.		
Morimura, 2011	Male	66	Anogenital	Genitourinary	1	Asymptomatic subcutaneous mass on the left side of the abdomen.	3 weeks	N	Not Listed.	Not Listed.	Although hidradenoma papilliferum in anogenital region was exclusively found in women, ectopic hidradenoma papilliferum was frequently found in men.	
Gatti, 2012	Male	79	Leg	Lower extremity	Nodule on his left leg that has been rapidly increasing in size.	6 months		Dark bluish, well-circumscribed, firm, non-fluctuant, mildly tender; non-painful, non-itchy, no bleeding.	Not Listed.	Histology- lobulated benign adnexal tumor derived from distal excretory sweat duct with a prominent clear cell component and diagnosis of eccrine acrospiroma was made.		
Duhan, 2011	Female	25	External genitalia	Genitourinary	8x6	Painless swelling of increasing size of the external genitalia.	4 months	N	Pedunculated, non-tender, soft swelling arising from the sulcus between L sided labium majora and minora, at the junction of upper 3/4th and lower 1/4th.	Not Listed.	Disease-free 2 years.	
Shah, 2010	Male	30	Scrotum	Genitourinary	Not Listed.		Not Listed.	Not Listed.	Not Listed.			
Rubenzik, 2010	Male	83	Nose	Face		Not Listed.		Not Listed.	Not Listed.	Not Listed.		
Novak, 2010	Male	70	Chest	Chest	2.5diameter	Subcutaneous nodular lesion on the right side of the chest beneath the breast.	6 months		Red-colored, exulcerated tumor with serous-bloody discharge on the tumor surface.	Not Listed.	Not Listed.	
Gonul, 2010	Female	9	Leg	Lower extremity	3.5x2	Gradually enlarged and ulcerated nodule on the left leg.	1 year	N	Firm, non-tender erythematous tumoral lesion on the left leg.	Not Listed.	Histology- circumscribed tumor centered in dermis connected with epidermis; clear and polyhedral cells; cystic spaces and few duct-like structures.	
Rekhi, 2011	Female	26	Leg	Lower extremity	3x3	Pain and swelling in the left thigh x 10 days.	10 days	N	Non-tender, soft tissue mass.	Not Listed.	Histology- cellular with cells arranged in cohesive clusters, tubular arrangements, and scattered singly; cells were polygonal with scanty to moderate amount of blue cytoplasm and focally displayed fi	
Cho, 2010	Female	56	Axilla	Upper extremity	2.7x2.8	Solitary, palpable mass in the left axilla with rapid growth.	6 months		Not Listed.	Not Listed.	Histology- clear cells with ductal differentiation and eosinophilic cytoplasm.	
Lee, 2010	F, F	66, 46	Nose, Nose	Face, Face	0.7x0.7.1.0	Slowly growing asymptomatic lesion on the nose. Asymptomatic nasal cutaneous lesion.	Not Listed, Not Listed	Not Listed, Not Listed	Soft dome-shaped papule with overlying skin. Well-demarcated, slightly erythematous, dome-shaped nodule with central ulcerations.	Not Listed, Not listed.	Not Listed, Not Listed.	Not Listed, Not Listed.
Jariwala, 2010	Male	53	Right foot	Lower extremity	1.0diameter	Chronic non-healing ulcer attributed to an injury 4 years prior when toe became stuck in the bath tap.	4 years		Dusky, reddish blue in color.	Not Listed.	Not Listed.	
Garcia-Bonafe, 2009	Female	60	Scalp	Head		Not Listed.	6 months		Not Listed.	Not Listed.	Histology-lobulated pattern with necrosis and cyst formation; clear cells seen on cytology occupied periphery of the lobules, whereas anaplastic cells were located in central portion; squamous differe	
Moon, 2009	Female	70	Scalp	Head	5x4.5x4sized	Large cystic mass on scalp, slowly-growing, asymptomatic.	3 years		Heart-shaped mass, dusky-bluish cystic nodule with marked telangiectatic surface and soft consistency like a jelly.	Not Listed.	Not Listed.	
Sarma, 2009	Male, Male	75, 60	Hip, Buttock	Pelvis, Lower extremity	1,0.5	Dome-shaped red papule on right hip. Left buttock nodule.	Not Listed, Not Listed	Not Listed, Not Listed	Not Listed, Not Listed.	Not Listed, Not Listed.	Not Listed, Not Listed	Not Listed, Not Listed.
Yu, 2010	Male	43	Knee	Lower extremity	8.0x6.0x3.0	Not Listed.		Mobile.	Not Listed.	Not Listed.		
Brahim, 2010	Male	75	Shoulder	Upper extremity	6-year history of recurrent mass that was removed 2 years ago.	Not Listed.	Not Listed.	Not Listed.				
Yildiz, 2009	Female	41	Glutes	Lower extremity	Not Listed.		Not Listed.	Not Listed.	Histology-eosinophilic cytoplasm, uniform-appearance, oval-round-nucleus, benign tumor with cystic and solid components.			
Battistella, 2010	Female, Male	43, 64	Scalp, Left shoulder	Scalp, Upper extremity	NotListed,NotListed	Not Listed, Not Listed.	Not Listed, Not Listed	Not Listed, Not Listed	Not Listed, Not Listed.	2 successive incomplete resections. Not Listed.	Not Listed, Not Listed	Discussed treatment with Sunitinib. Discussed treatment with Sunitinib.
Wahl, 2009	Male	54	Scalp	Head	1	Solitary, erythematous, rapidly growing 1 cm nodule on his scalp that has arisen over the past 3 months.	3 months		Not Listed.	Not Listed.	Tumor cells focally involved overlying epidermis - Paget's disease.	
Gupta, 2009	Male	12	Forehead	Face	4x3	Gradually increasing painless nodule on the forehead for 8 months.	Not Listed.	Not Listed.	Not Listed.			
Al-Qattan, 2009	Male	55	Left thumb	Upper extremity	Not Listed.		Not Listed.	Not Listed.	Dx: benign eccrine poroma.			
Veeranna, 2009	Female	35	Vulva	Genitourinary	1x1	Asymptomatic solitary lesion on the labia majora for 6 months.	6 months	N	Solitary nodule, eroded surface.	Initially small swelling and gradually increased in size.	Not Listed.	
Yu, 2009	Male	86	Ankle	Lower extremity	Not Listed.		Not Listed.	Not Listed.	Not Listed.			
Rongioletti, 2009	Male	94	Back	Back		Not Listed.		Not Listed.	Not Listed.	Squamomelanocytic tumor. Could be hidradenoma colonized by melanocytic maligt component.		
Tanese, 2010	Male	86	Hand	Upper extremity	Enlarging tumor on the dorsum of the left hand.	Not Listed.	Not Listed.	Maligt eccrine spiradenoma. No signs of recurrence or metastasis in the 2 years of follow-up after excisional biopsy.				
Driscoll, 2009	Male	37	Groin	Genitourinary	Quarter-sizedskinlesion	Right inguinal mass that has progressively increased in size over the previous months.	Previous months	Not Listed.	Had same skin lesion 11 years earlier and dx as hidradenoma that extended to the surgical margins with recommendation for complete excision. Shortly after biopsy, mass recurred, elevating surrounding skin and tripled in size. Lesion was stable then lost to follow-up.	Not Listed.		
Yoshida, 2008	Female	88	Knee	Lower extremity	1.1x0.8	Right knee nodule, enlarging gradually, with occasional bleeding.	Dome-shaped, flesh-colored nodule with ulceration on its surface.	Not Listed.	Histology- solid tumor cell nests with abundant cystic spaces or ductal structures. In solid nests, many clear cells and some slightly dark cells were seen. Most cystic spaces contained homogenous eos			
Fraedrich, 2008	Male	61	Cheek	Face		Huge mass on left cheek extending to the parotid gland, causing peripheral facial nerve palsy.	Not Listed.	Not Listed.	Not Listed.			
Jain, 2008	M	70	Eye	Head	0.6diameter	Nodule and the loss of eyelashes.	3 months	N	Loss of eyelashes with node.	3 months from the first notice of a node; Mohs surgery for removal; follow-up in 6 months shows no recurrence.	Y	5 eyelid cases were reported, and they were found to predomitly older men (1:4).
Chiu, 2007	F	55	Back	Back	1.5x2.0	Brown, soft.	7 years	N	Brown soft, irregularly shaped nodule with blackish protruding papules.	Asymptomatic nodule for 7 years, and removed through excision.	N	Case of pigmented poroma, which is uncommon; contained brown fluid; prediction that pigmented eccrine poroma are more common in Asians.
Navi, 2008	M	65	Chest, Ankle, Forearm	Chest, Foot, Arm	NotLIsted	Lesions would bleed when traumatized and have persisted for several months.	Several months	Y	Flesh colored papules.	Lesions were present for several months, and were shaved to be removed.	N	Case of poroma with multiple locations with a total of 7, but only two are noted here since they definitively diagnosed 3.
Iwasaki, 2007	M	91	Eye	Head	0.4x0.5	1 year of painless slow growing nodule.	1 year	N	Reddish-pink and ulcerative.	Present for 1 year.	N	Closely resembles basal cell carcinoma.
Rabady, 2008	F	71	Eye	Head	0.5diameter	Right lower eyelid paresthesia and right eye tearing.	N	Flesh -coloured smooth surface papule.	Presented with papule, and excisional biopsy was performed; 12 months follow-up no recurrence.	N	Not Listed.	
Biedrzycki, 2008	F	39	Vulva	Genitourinary	Presumed left sided Bartholin Cyst.	10 months	N	Enlarged node.	7 months cyst; Post removal of cyst, 10 months later, enlarged left-sided groin nodes containing MCCH; re-excised 3 months later; 10 months follow-up no recurrence.	N	Clear cell hidradenoma found in a rare location. The removal of clear cell hidradenoma had residual tumor cells, resulting in maligt clear cell hidradenoma ten months later.	
Yaghoobi, 2007	M	14	Shoulder	Upper extremity	1.0x0.5	Firm, nontender erythematous nodule on left shoulder with an ulcerated surface.	1 year		Firm, nontender erythematous nodule on left shoulder with an ulcerated surface.	Progressively enlarging over 1 year.	N	CCH are usually not found in this decade of life.
Angulo, 2007	M, F	47, 78	Back, Arm	Back, Upper extremity	NotListed,2.0diameter	Nodular lesion present for 3 years. 2 year tender nodule (firm nodule covered by normal skin).	3 years	Not Listed, No.	Nodular lesion. Firm nodule covered by normal skin.	Nodular lesion on the center of patient back for 3 years, and was removed through excision. Nodule of 2 years on skin.	N, N	Instead of the usual clear or polygonal cells being predomit, these cases are predomitly squamous cells. Not Listed.
Collman, 2007	M	55	Ankle	Lower extremity	8.7x7.1x4.4	Large mass on medial aspect of right ankle.	14 years	Y	Large mass on medial aspect of right ankle.	Slow steady growth w/ intermittent fluctuations in size after injury with hammer 14 years previous. Throbs at night. Tense at anterior aspect with subsequent rupture. Watery, light to dark brown fluid drain every 2-3 weeks to reduce symptoms of pressure.	N	Large mass and first reported clear cell hidradenoma case on the ankle.
Ohi, 2007	F	55	Breast	Chest	0.8diameter	No complaint: she came in for a follow-up.	N	N/A.	Not Listed.	N	Not Listed.	
Goh, 2006	F, M, M	72, 40, 32	Eyelid, Shoulder, Scalp	Head, Upper extremity, Head	1.3,2.5,3	Not Listed, Not Listed, Not Listed.	Not listed, 6 months, Not listed	Not Listed, Not Listed, Not Listed	Not Listed, Not Listed, Not Listed	After excision, no recurrence after 4 year follow up. After excision, no recurrence after 3 year follow up. After excision, no recurrence after 4 year follow up.	N, N, N	Not Listed, Not Listed, Not Listed
Nash, 2007	M	44	Chest	Chest	1.2x1.1	Painful enlarging nodule in right chest for 3 months.	3 months	Y	Enlarging nodule.	Nodule 3 months duration at a location which a 'benign' nodule has been 8 years prior; palpable nodes in right axilla.	Y	Not Listed.
Knoedler, 2007	F	26	Breast	Chest	8.5x8.0x6.0	Rapid growth in the last 4 months of the 8 months since the lump was noticed; weeks prior to visit, two focal skin breakdown causing clear fluid to come out.	8 months	Y	Two focal sites of skin breakdown which wept clear fluid.	Rapid growth for the last 4 months of the 8 months since noticed.	N	Not Listed.
Liapakis, 2006	M, M	68, 45	Abdominal Wall, Axillary	Abdomen, Upper extremity	Notlisted,2.0diameter	Multiple satellite nodules development. Not Listed	Not Listed, Not Listed	Not Listed, Not Listed	Ulcerous lesion on the anterior surface. Whitish lump	Removal of eccrine spiradenoma leading to multiple satellite nodules which also contained CCH; patient died 9 months later from disseminated disease. Patient was admitted to hospital for complete wide resection and radical axillary lymphadenectomy; no residual disease or nodal metastases; 6 months follow-up no recurrence.	Y, N	Case of eccrine spiradenoma of sweat glands with squamous differentiation and intense desmoplastic reaction found using histology and CCH presence was found arising in sweat glands of nodal metastases
Gilaberte, 2006	F	1 (18 months)	Leg	Lower extremity	2.0diameter	N/A.	13 months	Brownish-red nodule with ulcerated center.	First noticed 13 months prior to removal of lesion; 13 months follow-up no signs of recurrence.	N	Very few cases of CCH in first decade of life.	
Lee, 2006	M	53	Groin	Genitourinary	5.0x4.5x3.0	Soft tissue mass.	1 year	N	Not Listed.	20 years prior, similar soft tissue mass was incompletely removed from same site.	N	Not Listed.
Ozawa, 2005	M	5	Forearm	Upper extremity	1.3x0.9	Tumor with smooth, pale-red surface, and some dilated capillaries.	6 months		Tumor with smooth, pale-red surface, and some dilated capillaries.	Nodule began enlarging 6 months prior, and it was excised; 8 moths follow-up no recurrence.	N	First decade of life.
Volmar, 2005	M	59	Axillary	Upper extremity	2.0diameter	Bloody stool and generalized abdominal discomfort for 1 day (this was for his renal cell carcinoma).	Axillary region nodule was present for "several years"	N	rubbery, mobile, subcutaneous.	Present for two years.	N	Found in a man with renal cell carcinoma.
Ghai, 2004	F	77	Breast	Chest	2.0diameter	Several years	Soft, mobile, non-tender mass with bluish discoloration.	Present for several years.	N	Not Listed.		
Ohta, 2005	F	27	Head	Head	3.0x3.0	Asymptomatic scalp mass.	10 months	N	Firm, smoothly elevated nodule with overlying scalp skin on vertex.	10 months growth, removal; 24 months follow-up no recurrence.	N	Not Listed.
Harada, 2003	M	66	Forehead		4.0diameter	small, dark brown, 5mm papule on forehead with gradual thickening beneath the lesion to 4cm.	The papule that was noticed remained unchanged for 1 year, but there was gradual thickening beneath the lesion. A timeline was not mentioned.	Subcutaneous tumor was very hard and fixed to the frontal bone. The small papule on top of the subcutaneous tumor showed slight indentation.	Not Listed.	Y	Apparent association with a small pseudoepitheliomatous hyperplasia. This was a type of reactive hyperplasia that showed downward proliferation of the epidermis into the dermis. Diagnosis of maligt	
Reier, 2002	M	60	Left foot		2x1x1	Painful mass in mid plantar region of the left foot.	3 years	Y	tender, no induration or erythema evident.	3 months before excision, there was increasing pain and size.	Diagnosis of eccrine acrospiroma.	
Holden, 2002	M	80	Scalp		2x2	new-onset bloody discharge from lesion that has been slowly growing for 20 years; also developed a parotid mass.	20 years	Y	ulcerated, mobile, nontender scalp mass.	slowly growing for 20 years.	Y	Diagnosis of maligt eccrine acrospiroma. Histologically, this lesion had components of both benign and maligt neoplasm adjacent to each other. This patient had central scalp lesion as well as a
Sagi, 2002	F	66	Thumb		1.5diameter	Painless mass.	1 year	N	nontender, firm mass, covering skin appeared normal.	Not Listed.	At the time of the case, CCH had never been reported to be found on the hand.	
Punia, 2001	F	10	Scalp		8x6x2	Gradually enlarging swelling in scalp following trauma 6 years earlier.	6 years		6 x 6 cm swelling in the parietooccipital region with an irregular surface and margins fixed to the skin.	Not Listed.	diagnosed as eccrine acrospiroma.	
Morrell, 2001	F	15	Arm		0.8x0.5	Referred to Dermatology for lesion on her left arm.	2 years	N	firm, solitary multilobulated, pinkish to flesh-colored papule.	Not Listed.	diagnosed as CCH.	
Yildirim, 2000	F	50	Arm		6x8	Admitted for diagnosis and treatment of a nodule 6 x8 cm in size that began to enlarge recently at that time.	12 years	Y	Smooth, mobile, with deep purple/black color.	had it for 12 years but started to enlarge near the time of her admission.	Y	Maligt CCH.
Will, 2000	M	40	Foot		1diameter	Red nodule on right lateral foot; this nodule was previously excised; growth had rapidly recurred following excision the first time.	Y	Firm, reddish lesion.	Was initially excised but had recurred.	Due to incomplete excision the clear cell hidradenoma had recurred on the foot.		
Yashar, 2000	M	8	Behind left ear	1.5x0.9	referred for evaluation of a nodular growth behind the left ear.	4 months	Y	Well-defined, tender, dome-shaped, translucent, red blue.	4 months.		CCH.	
Agarwala, 1999	F		Eyelid			Not Listed.		Not Listed.	Not Listed.	Not Listed.		
De Toma, 2000	F	17				Not Listed.		Not Listed.	Not Listed.	Not Listed.		
Faulhaber, 2000	F	5	Chest		2.5x2.0	admitted with an asymptomatic brownish-red tumor on the chest about 12 cm below the right mamilla.	1.5 years		firm and well-circumscribed, partly atrophic, shiny surface and extensive vascularization (telangiectasia).	Not Listed.	At the time of diagnosis only one other case of hidradenoma was documented in the first decade of life.	
Biernat, 1999	F	78	Face		2.8x1.8x1.0	Patient noticed small papule 2 years ago that enlarged rapidly within the last 10 months before the diagnosis was made.	2 years		Soft and nontender.	2 years, rapidly growth within 10 months.	At the time of diagnosis this case was described as an unusual example of benign adnexal neoplasm with features of follicular and apocrine differentiation.	
Dzwierzynski, 1999	F	56	Foot			Not Listed.		Not Listed.	Not Listed.	Not Listed.		
Buise, 1999	F	67	Leg			Admitted for diabetes mellitus and large ulcerating tumor was incidentally found on right upper leg.	6 years	Y	Large ulcerating tumor.	Not Listed.	Diagnosed with advanced malignant clear cell hidradenoma.	
Wilhelmi, 1999	F	53	Scalp		2diameter	Referred to plastic surgery for a scalp lesion. Reported frequent trauma on brushing her hair.	3 years		solitary 2 cm tuberous mass, grapelike in contour with bluish pigmentation.	3 years.		atypical eccrine acrospiroma due to cellular atypia, abundant mitotic figures and proximity to margins.
Maldjian, 1999	M	48	Leg		5.0x5.5	Recurrent tumor of the right thigh.	2 years prior had 1.5 cm mass excised	Multilobulated, elevated the skin; well-defined cystic mass in the subcutaneous tissue.	Not Listed.	First reported MR imaging findings of histologically proven clear cell hidradenoma.		
Gianotti, 1997	F, M, F, F, F	45, 35, 28, 46, 34	Back, Neck, Scalp, Scapula, Back	Not Listed, Not Listed, Not Listed, Not Listed, Not Listed	2diameter,NotListed,NotListed,3diameter,2diameter	Large nodule on her back. Fast-growing brown nodule on his neck with the skin over the tumor occasionally eroding and bleeding. Large slow-growing dull red tumor on her scalp. Exophytic neoplasm on left scapula. Tumor present for 3 years and grown larger in last 6 months prior to diagnosis. Pain evoked by compression.	1 year, Not Listed, 3 years, Not Listed, 3 years.	Not Listed, Y, Y, Not Listed, Y	Dull red tumor, dome shaped and eroded. Brown nodule, occasionally shedding and bleeding. Had a solid and cystic component. Large, slow growing, exophytic, dull red tumor on scalp. Not Listed. Nodule 2 cm in size with pain evoked by compression.	Reached 2 cm in diameter in 1 year. Not Listed. Slow growing over 3 years. Not Listed. Present for 3 years and grown larger in last 6 months prior to diagnosis.	Not Listed. Not Listed. Not Listed. Not Listed. Not Listed	Not Listed. Not Listed. Not Listed. Not Listed. Not Listed.
Feldman, 1997	M	59	Foot		1x2x2	Lump on the fourth toe of right foot.	8 months	Y	swollen right 4th digit with bulbous distention laterally and plantarly at the pulp of toe. Ulceration measuring 0.5 x 0.5 cm. Serous drainage noted.	Not Listed.	CCH.	
Dumont, 1996	M	51	Arm		1.2x1.0	Presented with firm nodule on his left arm gradually increasing in size over the year.	1 year	Y	Firm, nontender, smooth, blue-black cystic nodule on the left forearm.	1 year.		Diagnosed as an eccrine acrospiroma, clear cell type.
Laws, 1996	M	81	Elbow		1.8x1.6	Actinically damaged skin evaluated for a 6-month history of slowly enlarging nodule on left elbow; denied history of trauma, pruritic or pain.	6 months	N	nontender, red blue, multiloculated, cystic nodule.	Not Listed.	Acrospiroma.	
Fitzgibbon, 1996	F	29	Left cheek		1.3x1	Swelling that had been growing.	several months	N	Not Listed.	Growth over a period of a few months.	No recurrence or metastasis after 12 months.	
Marsico, 1994	M	51	Left axilla		4x4	Clear material draining from area.	3 years	Y	Violaceous, well-circumscribed mass.	Slowly growing over 3 years.	Not Listed.	
Hampton, 1992	F	64	Right shoulder	1x1	Draining cyst.	18 months	Y	1x1 cm soft, irregular nodule on her right shoulder with a 4x4 mm protuberant, erythematous, moist papule on one edge.	Had cyst on right shoulder that was tender and nondraining that she had surgically removed 18 months prior. However, it never healed and began to leak clear fluid.	Not Listed.		
Kato, 1992	M		Cheek			Not Listed.		Not Listed.	Not Listed.	Within two months of the primary operation to remove it, growth of the tumor into the overlying epidermis recurred rapidly.		
Hunt, 1991	F	55	Anterior neck		Slow growing nodule.	6 months	N	Serous fluid could be expressed from a central skin opening.	Not Listed.	Adnexal tumor with features of both clear cell hidradenoma and mucinous syringometaplasia.		
Grossniklaus, 1991	M	46	Left lower eyelid		Enlarging mass.	2 months		Not Listed.	Not Listed.	Not Listed.		
Hmeidan, 1991	F	55	Left inguinal region	2.5	Not Listed.		Not Listed.	Not Listed.	Not Listed.			
Stagnone, 1990	F	76	Right long finger		Not Listed.		Not Listed.	Not Listed.	Not Listed.			
Jagannath, 1990	F	70	Right lower eyelid	1.25x1x0.5	Small nodule on right lower eyelid.	1 year	Y	ulcerative mass with rolled out edges. No induration or discharge.	Had an excisional biopsy done when patient first noticed it. Patient was symptom free for 3 months when she again noticed a nodular mass in the right lower lid at the site of previous surgery.	Not Listed.		
Schleicher, 1989	M	68	Forehead		2.8nodule	Forehead lesion that had gradually progressed in size over the previous 4 years.	4 years	N	Reddish nodule with a smooth surface.	Not Listed.	Not Listed.	
Schroeder, 1989	M	47	Lower lip			Not Listed.		Not Listed.	Not Listed.	Not Listed.		
Kazakis, 1988	M	72	Posterior trunk		Not Listed.		Not Listed.	Not Listed.	After removal of lesion, patient had occurrence of a bullous eruption.			
Tanino, 1984	F	33	Left parietal skin	4indiameter	rapid enlargement.		Not Listed.	Not Listed.	Not Listed.			
Ogilvie, 1982	M	19	Lateral aspect of right foot	Not Listed.		Not Listed.	Not Listed.	Metastasized to two bones of the foot.				
Fathizadeh, 1981	F	27	Lateral aspect of right forearm	2x2	enlarging nodule.	2 months	N	soft, pigmented, smooth surfaced, nonfluctuant, nontender, superficial fixed nodule.	Not Listed.	Not Listed.		
Haneveld, 1979	F	87	Nasal side of upper eyelid	Rapidly growing tumor.	Not Listed.	Not Listed.	Invaded the conjunctiva.					
Morain, 1979	M	92	Nasal tumor	6x3.5x3	Not Listed.	25 years	N	Pedunculated mass, solid-cystic with a smooth multinodular configuration and a variegated blue grey to pink color.	Not Listed.	Not Listed.		
Dogan, 2017	M	14	Left inner thigh	10x7x12	painful mass.	2 months	Y	Blueish to skin colored soft tissue mass with multinodular appearance.	Started as a blueish red slightly tender plaque 2 months ago, thereafter it started to get firm and more appreciable. Soon after the mass grew to its final dimensions.	First reported case located on the thigh in childhood.		
